# Environmental risk factors for schizophrenia and bipolar disorder from childhood to diagnosis: a Swedish nested case–control study

**DOI:** 10.1017/S0033291724000266

**Published:** 2024-03-01

**Authors:** Natassia Robinson, Alexander Ploner, Marica Leone, Paul Lichtenstein, Kenneth S. Kendler, Sarah E. Bergen

**Affiliations:** 1 Department of Medical Epidemiology and Biostatistics, Karolinska Institutet, Stockholm, Sweden; 2 Janssen Pharmaceutical Companies of Johnson & Johnson, Solna, Sweden; 3 Department of Psychiatry, Virginia Institute for Psychiatric and Behavioral Genetics, Virginia Commonwealth University, Richmond, VA, USA

**Keywords:** adversity, bipolar disorder, environmental risk, schizophrenia, substance use

## Abstract

**Background:.:**

Shared genetic risk between schizophrenia (SCZ) and bipolar disorder (BD) is well-established, yet the extent to which they share environmental risk factors remains unclear. We compare the associations between environmental exposures during childhood/prior to disorder onset with the risk of developing SCZ and BD.

**Methods:.:**

We conducted a Swedish register-based nested case–control study using 4184 SCZ cases and 18 681 BD cases diagnosed 1988–2013. Cases were matched to five controls by birth year, birth region, and sex. Conditional logistic regression was used to estimate incidence rate ratios (IRR) for SCZ and BD for each exposure (severe childhood infections, adverse childhood experiences (ACEs), substance use disorders (SUDs), urban birth/longest residence).

**Results:.:**

All SUD types were associated with very high risk (IRR 4.9–25.5), and all forms of ACEs with higher risk (IRR 1.5–4.3) for both disorders. In the mutually adjusted models, ACEs demonstrated slightly higher risk for BD (SCZ IRR 1.30, 1.19–1.42; BD IRR 1.49, 1.44–1.55), while for SUD, risk was higher for SCZ (SCZ IRR 9.43, 8.15–10.92; BD IRR 5.50, 5.15–5.88). Infections were associated with increased risk of BD (IRR 1.21, 1.17–1.26) but not SCZ. Urban birth and urban longest residence were associated with higher risk of SCZ (IRR 1.19, 1.03–1.37), while only the combination of urban birth and rural longest residence showed higher risk for BD (IRR 1.24, 1.13–1.35).

**Conclusions:.:**

There were both shared and unique environmental risk factors: SUDs and ACEs were risk factors for both disorders, while infections were more strongly associated with BD and urbanicity with SCZ.

## Introduction

Genetic risk between schizophrenia (SCZ) and bipolar disorder (BD) overlaps considerably (Bulik-Sullivan et al., 2015; Lichtenstein et al., 2009). However, the 15–40% of risk stemming from environmental sources (Smoller & Finn, 2003; Sullivan, Kendler, & Neale, 2003) has received less attention and reflects a significant and potentially modifiable avenue of exploration.

Numerous environmental exposures have been implicated in SCZ, and there is convincing evidence for several infectious agents, adverse childhood experiences (ACEs), cannabis use and urbanicity, while evidence for associations with BD is much weaker (Robinson & Bergen, 2021). A wide range of infections have been associated with SCZ and BD, particularly central nervous system (CNS) infections (including toxoplasmosis) and influenza (Robinson & Bergen, 2021). Most studies have focused on the role of perinatal infections, and additional large studies on postnatal and childhood infections are needed. Studies examining the relationship between ACEs and SCZ and BD consistently report positive associations (Bailey et al., 2018; Matheson, Shepherd, Pinchbeck, Laurens, & Carr, 2013; Rowland & Marwaha, 2018); however, the majority of these studies are retrospective and few adjust for potential confounding factors. Strong evidence suggests a role of cannabis in the development of SCZ (Gage et al., 2017; Marconi, Di Forti, Lewis, Murray, & Vassos, 2016; Moore et al., 2007), and some support exists for this relationship with BD, too (Gibbs et al., 2015; Rowland & Marwaha, 2018). In general, substance use disorders (SUD) demonstrate high comorbidity with SCZ and BD. However, aside from cannabis, few studies have investigated specific types of SUD prior to onset of SCZ/BD. Increased risk of SCZ has been reported for people living in urban areas compared to more rural areas (Vassos, Pedersen, Murray, Collier, & Lewis, 2012), while findings for BD have been mixed (Kaymaz et al., 2006; Mortensen, Pedersen, Melbye, Mors, & Ewald, 2003; Vassos, Agerbo, Mors, & Pedersen, 2016).

These risk factors are likely intertwined as, for instance, people who experience childhood trauma are more likely to use cannabis (Harley et al., 2010). Spread of infections occurs more rapidly in urban, population dense areas (Neiderud, 2015). Access to drugs can also vary: in Sweden, illicit drug use is most common in urban areas (The Swedish Council for Information on Alcohol and Other Drugs (CAN), 2017). Despite separate investigations into the impact of these environmental factors on SCZ and BD, simultaneous and comprehensive assessment of the effects of these exposures and their individual contributions to the development of these disorders is lacking. Investigations into sex-specific effects are also limited; SCZ is more commonly diagnosed in males, and BD in females, yet whether associations between environmental risk factors and psychiatric diagnoses vary by sex remains largely unexplored. Following our previous study on pre- and perinatal risk factors (Robinson et al., 2023), and to gain a more comprehensive understanding of environmental risk factors over the life course, here we investigate postnatal environmental factors occurring between birth and diagnosis.

In this large, register-based study, we performed parallel investigation of several childhood and pre-onset environmental risk factors for SCZ and BD to determine: (1) whether risk associated with these exposures differs between the disorders, (2) whether the associations are independent of the other investigated environmental risk factors, and (3) whether risk varies by sex.

## Methods

### Study design and data sources

We conducted a register-based, nested case–control study among all persons born in Sweden between 1973 and 1998. We used data from several Swedish population-based registers linked via unique personal identity numbers. The Total Population Register (TPR), containing demographic data on all individuals resident in Sweden, was used to identify the study population (Ludvigsson et al., 2016). The National Patient Register (NPR), recording inpatient care in Sweden since 1964 (psychiatric diagnoses from 1973), and outpatient visits since 2001 (Ludvigsson et al., 2011), was used to identify SCZ and BD cases. We used several registers to define our exposure variables (Table 1 and online Supplementary Table S1–4). This study was approved by the Regional Ethics Review Board in Stockholm, Sweden (DNR 2013/862–31/5), and did not require informed consent due to analysis of pseudo-anonymized register data.

**Figure 1.**
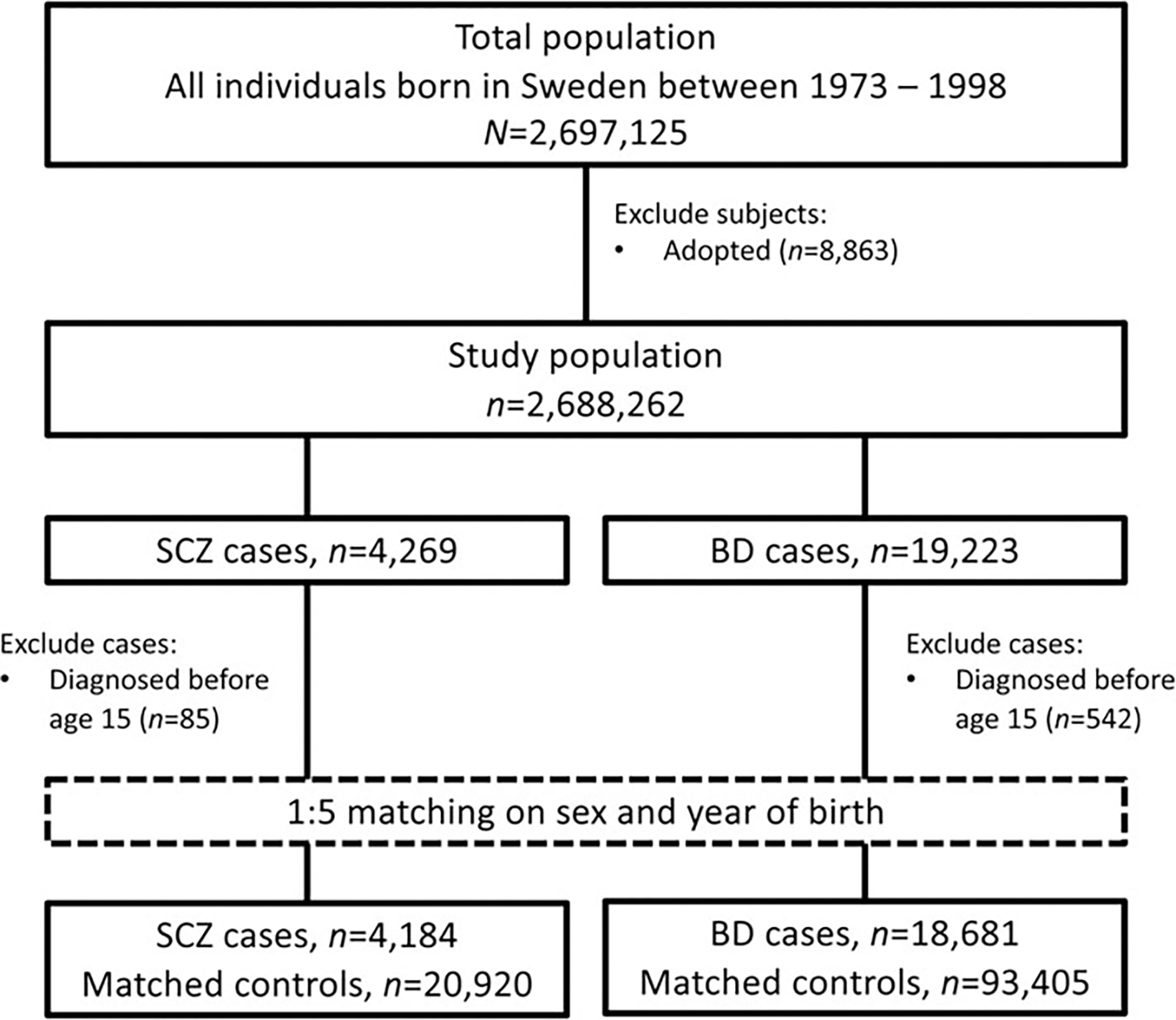
Flow chart of study population selection.

All cases of SCZ and BD were selected at their first diagnosis recorded in the NPR. In addition to the clinical definitions described under Outcomes, cases were required to be ≥15 years old at diagnosis. Individuals were eligible for inclusion if they had no prior history of emigration from Sweden in the Migration Register (Ludvigsson et al., 2016) at time of sampling; adopted individuals were excluded (Fig. 1). For each case, we identified five controls from the TPR matched on birth year, sex, and birthplace (county) and without SCZ or BD diagnosis, respectively, at the date of diagnosis of their matched case (i.e. incidence density sampling).

### Assessment of outcomes

We defined diagnoses in the NPR with International Classification of Diseases, Ninth Revision (ICD-9, 1987–1996), and International Statistical Classification of Diseases and Related Health Problems, Tenth Revision (ICD-10, 1997–2013) codes for SCZ (295 and F20, excluding simple, acute, latent and schizoaffective types) and BD (296 and F30–31, excluding unipolar melancholic, other and unspecified types) (online Supplementary methods). We identified inpatient and outpatient diagnoses in the NPR between 1 January 1988 and 31 December 2013. SCZ and BD were diagnosed using a non-hierarchical diagnostic structure, meaning individuals with both diagnoses contribute to analyses of both disorders (*n* = 425).

### Assessment of exposures and covariates

Record of any infection, any ACE and any type of substance abuse were primary exposures. Infection was defined by inpatient or outpatient clinical contact for any infection before age 15. ACEs encompassed abuse, neglect and household dysfunction (Cronholm et al., 2015) before age 15 (Table 1). SUD included any SUD diagnosis at least two years prior to SCZ/BD diagnosis or sampling date. To explore potential factors underlying these main exposures, we also examined risks associated with contributing sub-exposures (Table 1, online Supplementary Methods).

Urbanicity exposure was based on population density categories defined according to Eurostat guidelines (European Commission & Eurostat, 2019), applied to Swedish municipalities. Participants were classified based on population density of their municipality (i) at birth, and (ii) of longest residence prior to SCZ/BD diagnosis or sampling date, capturing complementary aspects of urban exposure (early life v. later/cumulative).

A potential confounder, childhood socioeconomic status (SES), was determined from highest achieved parental education and quartiles of household disposable income at age 15.

### Statistical analysis

Descriptive statistics are presented as counts and percentages by case–control status and by sex. Conditional logistic regression was used to calculate odds ratios, interpreted as incidence rate ratios (IRR), for risk of SCZ and BD in relation to exposures. IRR with 95% confidence intervals (95%CI) are presented relative to the respective unexposed reference group. We present findings for the main exposures (any infection, any ACE, any SUD, urbanicity), models mutually adjusted for each main exposure and SES to estimate the risk independent of the other factors and potential confounders (presented as adjusted IRR, aIRR), and models for each sub-exposure to determine their individual risk estimate. For sub-exposures, *p*-values were corrected for multiple testing using Benjamini–Hochberg false discovery rate (FDR). All analyses were also conducted stratified by sex. Two-sided Wald tests were used to evaluate differences between SCZ and BD risk estimates, and to evaluate differences between male and female risk estimates. Statistical analyses were conducted in R (version 4.0.5) (R Core Team, 2013), using the Survival package (version 3.2–10) for regression analyses (Therneau & Lumley, 2014).

## Results

We identified 4184 SCZ cases and 18 681 BD cases during our study period (Table 2) which is in accordance with prior Swedish studies (Dykxhoorn et al., 2019; Kendler, Ohlsson, Sundquist, & Sundquist, 2021; Sariaslan et al., 2014). Most SCZ cases were male (66%), and most BD cases female (66%). Median age at diagnosis was 25.0 for SCZ (IQR 21.5–29.2) and 25.4 for BD (IQR 21.2–30.6). Exposure frequencies in SCZ and BD cases and controls are summarized in Table 2. Most exposures were more prevalent in SCZ and BD cases v. their respective controls (for both sexes, online Supplementary Table S5), including any infection, any ACE, all individual ACEs, and all forms of SUD (Table 2). The main environmental exposures did not demonstrate strong correlations (r ≤ 0.1; Supplemental Figure S1).

### Risk associated with the main exposures

**Figure 2.**
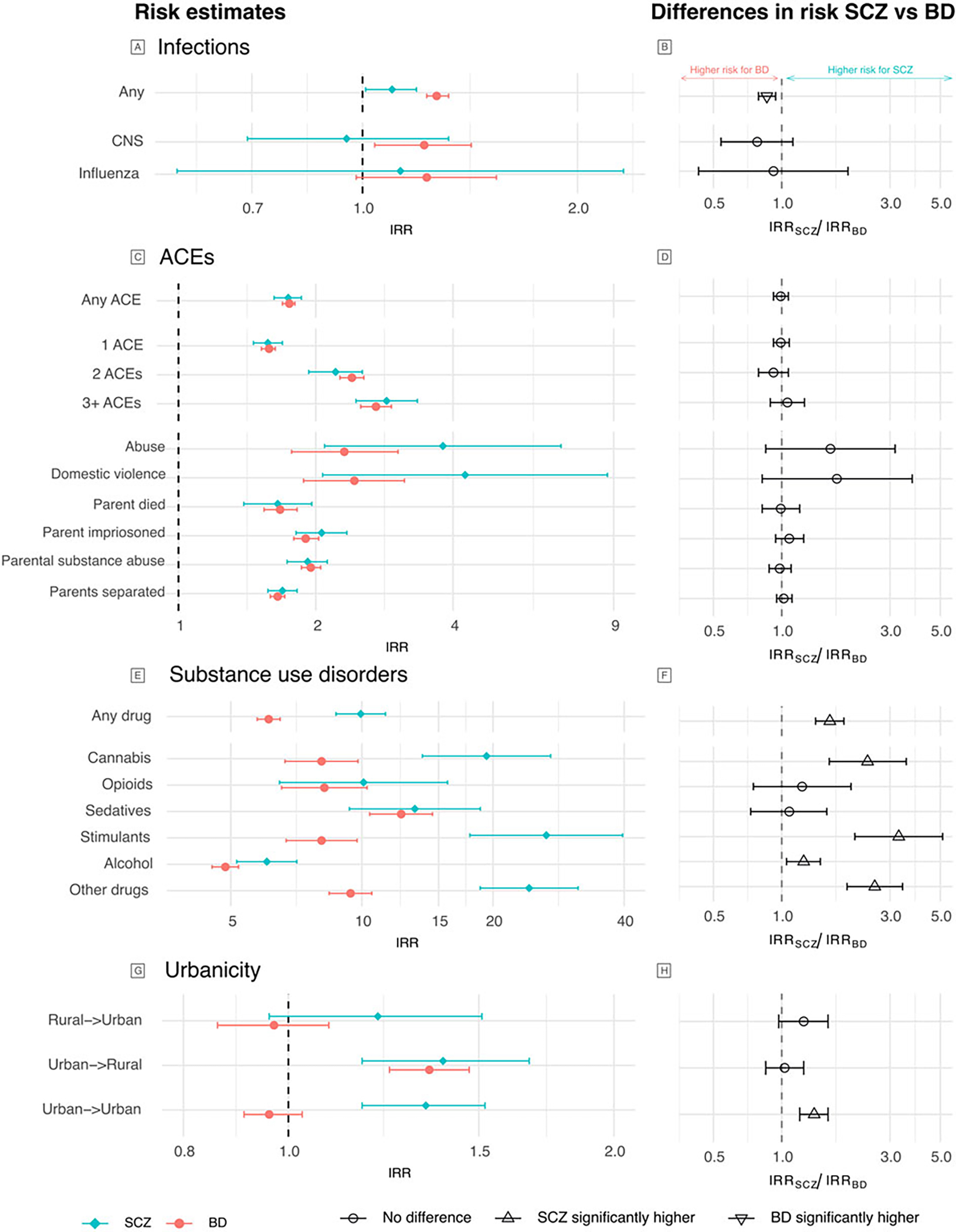
IRR for the individual environmental exposures and risk of SCZ and BD. Panels A, C, E, G show the unadjusted risk estimates for variables within each exposure category, and panels B, D, F, H show the comparison of the effects between SCZ and BD using Wald tests. Estimates greater than 1 are higher in SCZ and lower than 1 are higher in BD. Axes are on the log scale. Results can also be found in Table S6.

Table 3 shows the unadjusted IRR for the main environmental exposures and the mutually adjusted IRR (aIRR), including all factors and childhood SES. Diagnosis of any childhood infection was associated with a small increase in risk of SCZ (IRR 1.10, 95% CI 1.01–1.19,) and statistically significantly higher risk of BD (IRR 1.27, 95%CI 1.23–1.32, Wald test *p* = 0.001) (Fig. 2A–B). Experiencing any ACE was associated with similarly increased risk of SCZ (IRR 1.74, 95%CI 1.62–1.86) and BD (IRR 1.75, 95%CI 1.69–1.80, Wald test *p* = 0.89, Fig. 2C–D). Diagnosis of any SUD was associated with increased risk of SCZ (IRR 9.94, 95%CI 8.72–11.33) and to a lesser extent BD (IRR 6.11, 95%CI 5.75–6.49, Wald test *p* < 0.005, Fig. 2E–F).

Compared to the reference of rural birth and rural longest residence, urban birth was associated with a statistically significantly increased risk of SCZ, regardless of longest residence (with rural longest residence: IRR 1.39, 95%CI 1.17–1.67; with urban longest residence: IRR 1.34, 95%CI 1.17–1.52, Fig. 2G); rural birth with longest urban residence was associated with a smaller and statistically non-significant increased risk of SCZ (IRR 1.21, 95%CI 0.96–1.51, p_FDR_ = 0.11). In contrast, for BD, only the combination of urban birth and longest rural residence was associated with a statistically significantly increased risk (IRR 1.35, 95%CI 1.24–1.47, Fig. 2G). Comparing excess risk between SCZ and BD, we found a statistically significantly greater risk of SCZ for urban birth/urban residence (IRR ratio 1.40, 95%CI 1.21–1.61, Fig. 2H).

Compared to the unadjusted models, IRRs in the adjusted models were generally attenuated for both outcomes (Table 3); exceptions were the IRRs for BD involving longest urban residence, where the minimal protective effects of the unadjusted models were amplified to ~10% reduced risk (with rural birth: IRR 0.90, 95% CI 0.79–1.02; with urban birth: IRR 0.91, 95% CI 0.85–0.98). Statistical significance of the IRRs was somewhat diluted for SCZ, with infections no longer nominally statistically significant; in contrast, statistical significance of IRRs at the 0.001-level was preserved for BD as the outcome. Although there was no evidence of differences in risk in the unadjusted models, after adjustment, any ACE was associated with marginally yet significantly higher risk of BD than SCZ (SCZ IRR 1.30, 95% CI 1.19–1.42; BD IRR 1.49, 95%CI 1.44–1.55).

### Risk associated with the individual environmental factors

When considering infection type, for BD only, there was increased risk for CNS infections (IRR 1.22, 95%CI 1.04–1.42), and a non-significant (p_FDR_ = 0.09) increased risk for influenza infection (IRR 1.23, 95%CI 0.98–1.54).

Cumulative exposure to ACEs was associated with increasing risk of both disorders (online Supplementary Table S6). All individual ACEs were associated with increased risk for both disorders (IRR 1.7–4.3), with the highest risk for domestic violence and abuse, despite more statistical uncertainty due to low prevalence. We observed no difference in risk associated with ACEs between disorders (Wald test *p* > 0.1, Fig. 2D).

In fact, most types of SUD demonstrated significantly higher risk for SCZ (IRR 6.1–26.5) than BD (IRR 4.9–12.3, Fig. 2F). The highest risk for SCZ was evident in those with stimulant (IRR 26.51, 95%CI 17.70–39.69) or cannabis (IRR 19.33, 95%CI 13.77–27.13) use disorders, while for BD, the greatest risk was in those with sedative use disorder (IRR 12.31, 95%CI 10.42–14.53). Risk associated with opioid or sedative abuse was comparable between the disorders, while all other forms of SUD were associated with significantly higher risk of SCZ. Comparatively lower risk was observed for alcohol abuse (SCZ IRR 6.05, 95% CI 5.16–7.09; BD IRR 4.86, 95%CI 4.53–5.21).

### Sex differences in environmental exposures and risk of SCZ and BD

There were sex differences in the relationship between some environmental risk factors and our outcomes (online Supplementary Table S7–S8). Any form of substance abuse was associated with much higher risk of SCZ in males, even after adjustment (aIRR 10.78, 95%CI 9.00–12.93, v. female aIRR 7.25, 95%CI 5.63– 9.35), and risk associated with stimulants was over twice as high in males than females (IRR 37 *v*. IRR 15); however, the prevalence of specific types of SUD was low in the sex-stratified analysis (online Supplementary Table S5). Parental substance abuse was associated with marginally higher risk of SCZ in males (male IRR 2.07; female IRR 1.64, *p* = 0.04), and marginally higher risk of BD in females (male IRR 1.83; female IRR 2.01, Wald-test *p* = 0.07).

For BD, females had marginally higher risks for any infection (aIRR 1.25, 95% CI 1.19–1.32; *v*. males aIRR 1.14, 95%CI 1.06–1.22). For any form of SUD, the risk of BD was slightly higher for males (aIRR 6.05, 95%CI 5.43–6.75) than females (aIRR 5.24, 95%CI 4.82–5.69), yet for sedative use disorder specifically, the risk was distinctly higher for females (female IRR 14, *v*. male IRR 10).

## Discussion

By concurrently investigating several environmental risk factors, our study provides new insights into their differential impact on risk of SCZ and BD. We found that experiencing any ACE and having any SUD significantly increases the risk for both disorders, however, ACEs show a slightly stronger association with BD, whereas SUD posed a higher risk for SCZ. The risks associated with individual ACEs were consistent for both disorders, while the risk associated with types of SUD varied by disorder. Urban birth or residence was associated with increased risk of SCZ, but only the combination of urban birth and rural residence was a risk factor for BD. Additionally, infections exhibited a stronger association with BD.

### Substance use disorders

We found that all forms of substance use disorder, not just cannabis, were associated with substantially increased risk of SCZ and BD. Associations across all types of substances may be due to direct causal effects on risk, shared genetic predispositions, or other shared risk factors (Agrawal, Neale, Prescott, & Kendler, 2004; Khokhar, Dwiel, Henricks, Doucette, & Green, 2018). Apart from opioids and sedatives, which demonstrated similarly increased risk for either disorder, risk associated with the other SUDs was higher for SCZ, implying that the nature of the association with SCZ and BD differs. Our estimates were higher than in previous Swedish studies, which may be due to differences in exposure measurement: Giordano, Ohlsson, Sundquist, Sundquist, and Kendler (2015) used diagnoses *and* criminal convictions for drug possession, and Zammit, Allebeck, Andreasson, Lundberg, and Lewis (2002) used self-reported data in military conscripts, both of which likely capture more recreational use. In contrast, our definition involved drug/alcohol use eliciting medical attention.

We found that SUD is associated with elevated risk of both disorders regardless of gender, but it may be a stronger risk factor for SCZ in males than in females, particularly for stimulant use. Although SUD was also associated with slightly higher risk for BD in males, the risk associated with sedative use disorder was much higher for females. These estimates should be interpretated with caution as they were relatively rare exposures. Studies frequently report sex differences in substance use generally and have also identified higher rates of SUD in males with SCZ, compared to females, and compared to males with BD; however, the majority examine comorbid substance use (Cantor-Graae, Nordström, & McNeil, 2001; Nesvåg et al., 2015). Sex differences in SUD in relation to SCZ and BD may arise from sex differences in behavior and in response to specific symptoms (Becker, McClellan, & Reed, 2017). For example, young men tend to engage in risky behaviors and experiment with drugs, while females may be more likely to use drugs for self-medication to cope with stress and trauma-related symptoms (Becker et al., 2017). Additionally, biological differences and gender-specific barriers such as drug accessibility and socio-cultural influences may also impact substance type and other differences in addiction.

Several considerations affect the interpretation of our findings, including polysubstance use, familial confounding, and reverse causation. We examined SUD diagnoses independently, however a diagnosis of one form of SUD does not discount the use of other drugs. In fact, polydrug use is common – 56% of patients entering drug treatment centers in Sweden use more than one drug (EMCDDA, 2009). It is difficult to establish the direction of association between SUD and SCZ/BD, though there is evidence that SUD generally precedes the onset of SCZ (Giordano et al., 2015) and BD (Levin & Hennessy, 2004); and despite the two-year buffer period between SUD and SCZ/BD diagnoses in our analyses, our results should not be used to infer causality. Indeed, evidence from co-relative studies on SCZ risk suggests that associations for opiates and sedatives are likely non-causal (arising from familial confounding) (Giordano et al., 2015), while stimulants had a modest causal effect, and cannabis was likely partially causal (Gillespie & Kendler, 2020; Giordano et al., 2015; Vaucher et al., 2018). Cannabis is one of the most widely used psychoactive substances, but only a fraction of users develop SUD or SCZ/BD – suggesting there are other contributing factors. Further exploration of these potentially bidirectional relationships is needed to determine the causal mechanisms and identify people most at risk for developing these disorders following exposure to specific drugs.

### Urbanicity

In our study, we used a novel approach to operationalize urbanicity as a two-factorial exposure, separating urbanicity at birth and longest residence. We identified a qualitative difference in risk of SCZ and BD based on rural or urban longest residence. For SCZ, we found evidence for a consistently increased risk for urban birth, regardless of longest residence, while for BD, only the specific combination of urban birth/longest rural residence showed clearly elevated risk. Consequently, while it makes sense to consider ‘urban birth’ as a risk factor for SCZ independent of subsequent residence, this is not the case for BD.

Urban birth and upbringing have been consistently associated with ~2-fold increased risk of SCZ (Lewis, David, Andréasson, & Allebeck, 1992; Vassos et al., 2012). The comparatively lower risks in our study (IRR < 2) may be partly attributed to measuring urbanicity at the municipality level, which can be a rather crude proxy for a lived urban experience, and matching controls on birth county, which attenuates the strength of the association (while controlling for many other exposures). There have been only two well-powered studies of BD; one supported an association for urbanicity at birth (Vassos et al., 2016), while the other did not (Mortensen et al., 2003). There have been no large studies on urbanicity later in life (>500 cases) (Rodriguez et al., 2020). Our findings may also explain why earlier studies have yielded contradictory findings.

Potential explanations for our distinct findings for SCZ and BD could be differences in underlying genetic risk, or that urban/rural exposure operates via different causal mechanisms. Individuals with higher genetic risk for SCZ and BD preferentially move from rural environments to cities (Maxwell, Coleman, Breen, & Vassos, 2021), suggesting that genetic risk, indexed by polygenic risk scores, appears similar. If urban exposure is causal, then it must operate via different mechanisms; either urban environments have different influences on etiology or specific symptoms. Indeed, some evidence suggests that urbanicity may be more strongly correlated with psychotic symptoms (Kaymaz et al., 2006). While we utilized population density to determine urbanicity, it represents a proxy for several characteristics such as reduced social cohesion, limited green space, and more light and noise pollution, which might be particularly stressful for individuals with an increased liability for SCZ and BD (Gruebner et al., 2017) and could explain the higher likelihood of BD cases to reside in rural areas. On the other hand, living in urban areas can also have health advantages, like improved access to healthcare, resources and large psychiatric facilities (Vlahov, Galea, & Freudenberg, 2005), which may be required more in management of SCZ (Jacobs et al., 2015). Urbanicity estimates were attenuated after adjustment for the other environmental exposures and SES, highlighting the intertwined relationships between these exposures. It is projected that over two thirds of the world’s population will live in urban areas by 2050 (UN, 2018), hence greater understanding of the precise mechanisms is needed.

### Adverse childhood experiences

We found similar patterns of increased risk of SCZ and BD across all ACEs studied. Our findings align with a previous study in young adults in Sweden, which found that childhood adversity increased risk across different psychiatric disorders (Björkenstam, Burström, Vinnerljung, & Kosidou, 2016). Meta-analyses find slightly higher risk than we observed (SCZ OR = 3.6, *n* = 464 SCZ cases (Matheson et al., 2013); BD OR = 2.6, *n* = 1259 BD cases (Palmier-Claus, Berry, Bucci, Mansell, & Varese, 2016)) and while we examine many more cases it is possible that our results underestimate the risk by using broad but objective measures of ACEs, as opposed to more detailed but retrospective methods such as questionnaire or surveys.

This study adds to the growing literature that the effects of ACEs are not disorder-specific, and increase risk for numerous psychiatric and somatic disorders (Bellis et al., 2019). Yet, any ACE was associated with marginally higher risk for BD than SCZ in the adjusted model, suggesting that the impact of the exposures differs by disorder. We found evidence of a dose-response effect, which has also been observed across several domains of mental and physical health (Anda et al., 2006; Mersky, Topitzes, & Reynolds, 2013). These expansive effects are due to the involvement of several neurobiological systems and stress-mediated mechanisms (Anda et al., 2006). ACEs are common across all populations, and interventions to minimize their impact would have beneficial impacts on rates of many disorders.

### Infections

We found evidence that severe childhood infections were associated with BD, even when controlling for other factors which reportedly increase the likelihood of infections, including social inequity and urban living (Neiderud, 2015; Pini et al., 2019; Semenza & Giesecke, 2008). A Danish register study found that hospital contacts for infections increased the risk of BD, although these effects extended to most mood disorders (Benros et al., 2013). This study, and another Swedish study (Leone et al., 2022) also observe slightly higher risk for mood disorders for any infection in females, which might suggest that these sex differences are a feature of mood disorders more broadly.

The association between childhood infections and SCZ was weaker, and statistically non-significant after adjustment for SES. This contrasts with a Danish register study, which found childhood infections requiring hospitalization were associated with subsequent SCZ, even after adjustment for SES (Debost et al., 2019); the study had proportionally more infections in cases and spanned different years than our study, which may account for these differences. It is possible that risk differs by infection type, with higher rates of SCZ in those exposed to viral, but not bacterial CNS infections in childhood; however, studies stratifying by type of infection have few exposed cases (Khandaker, Zimbron, Dalman, Lewis, & Jones, 2012). Our recent study of early life risk factors yielded contrasting results, whereby prenatal infections were associated with risk of SCZ but not BD (Robinson et al., 2023). Studies have suggested the possibility of distinct sensitive periods for infection exposure (Robinson & Bergen, 2021), therefore infection during early development, as opposed to in childhood, may have differential impacts on the underlying mechanisms of SCZ and BD. Further investigation is warranted to better understand these differences.

### Strengths and limitations

This large-scale national register study included comprehensive, almost total population coverage with minimal selection bias. Using registry data limits recall bias and misclassification, and we examined objective measures of ACEs supplemented with crime data. Matching on birth year accounts for period effects and temporal changes in diagnostic criteria. The validity of our findings is contingent on the quality of the SCZ and BD diagnoses in the Swedish national registries, which have been well demonstrated (Ekholm et al., 2005; Lichtenstein et al., 2006; Sellgren, Landén, Lichtenstein, Hultman, & Långström, 2011). Although surprising, the higher prevalence of BD compared to SCZ is consistent with prior Swedish studies (Dykxhoorn et al., 2019; Kendler et al., 2021; Sariaslan et al., 2014). There was a steep rise in documented BD cases in 2001 (Carlborg, Ferntoft, Thuresson, & Bodegard, 2015), which coincides with the start of the outpatient register.

Even with our large sample, some exposures were relatively rare, particularly for the sex-stratified analysis. After 2001, introduction of outpatient diagnoses increased coverage in the NPR; however, as these do not include primary care diagnoses, these likely capture more severe cases of infections and SUDs. We performed adjusted analysis to control for the effects of the main exposures and covariates, but further studies are required to address potential confounding by familial (environmental and genetic) factors which can also influence risk (Li et al., 2022).

## Conclusions and future directions

By identifying the parallel and disparate environmental risk factors for SCZ and BD, this study provides insight into the etiology of SCZ and BD. These results emphasize the importance of the potential impact of the childhood and adolescent environment on severe psychopathology in adults. The excess risk identified for all forms of SUD with both disorders challenges the narrow focus on cannabis in previous research, and suggests that a comprehensive approach that considers all forms of SUDs may have clinical utility in identifying high-risk individuals. The sex differences we identified should be regarded as preliminary findings that require further research. Additional studies are necessary to explore the mechanisms by which all these exposures confer risk and to better understand how urbanicity and infections differentially impact risk of SCZ and BD, as well as further exploration of the causal pathways and relationships between exposures.

## Supplementary Material

Supplement

## Figures and Tables

**Table 1.** Definitions of the environmental exposure variables

Exposure group	Main exposure	Sub-exposures	Exposure timing	Register
Infection	Any infection	Any central nervous system infection, any influenza infection	Age 0–15	NPR
Adverse childhood experiences (ACEs)	Any ACE (see individual ACE components)	Number of ACEs (0,1,2, 3+); individual ACEs (child abuse, domestic violence, parental separation, parental imprisonment, parental substance abuse, parental death)	Age 0–15	NPR, LISA, censuses, death, crime, and suspect registers.
Substance abuse	Diagnosis of any type of substance abuse	Individual diagnoses of substance use disorder for: cannabis, opioids, sedatives, stimulants, alcohol and other drugs	At least two years before SCZ or BD diagnosis	NPR
Urbanicity	Population density category of place of birth v. longest residence (Always rural, Rural −> urban, Urban −> rural, Always urban)		Before SCZ or BD diagnosis	LISA

Full descriptions of the variable definitions can be found in the supplementary methods. The variables were derived from several registers including the National Patient Register (NPR) which contains data on all inpatient diagnoses since 1973 and outpatient data since 2001; the Longitudinal integrated database for health insurance and labor market studies (LISA) databases and the Censuses (1960–90) contain information on family structure and socioeconomic factors; death register contains date of death; and the National Crime Register includes all criminal convictions since 1973.

**Table 2.** Descriptive characteristics of the environmental risk factors in the SCZ and BD samples

Characteristic	SCZ			BD		
	Overall, *N* = 25104	Control, *N* = 20 920	Case, *N* = 4184	Overall, *N* =112 086	Control, *N* = 93 405	Case, *N* =18 681

**Any infection**	5407 (22%)	4450 (21%)	957 (23%)	24 451 (22%)	19 724 (21%)	4727 (25%)

CNS infection	269 (1.1%)	226 (1.1%)	43 (1.0%)	1020 (0.9%)	821 (0.9%)	199 (1.1%)

Influenza	49 (0.2%)	40 (0.2%)	9 (0.2%)	468 (0.4%)	376 (0.4%)	92 (0.5%)

**Any ACE**	10 822 (43%)	8556 (41%)	2266 (54%)	49 964 (45%)	39 543 (42%)	10 421 (56%)

Number of ACEs						

*0*	14 282 (57%)	12 364 (59%)	1918 (46%)	62 122 (55%)	53 862 (58%)	8260 (44%)

*1*	8683 (35%)	7004 (33%)	1679 (40%)	40 288 (36%)	32 534 (35%)	7754 (42%)

*2*	1308 (5.2%)	977 (4.7%)	331 (7.9%)	6221 (5.6%)	4561 (4.9%)	1660 (8.9%)

*3+*	831 (3.3%)	575 (2.7%)	256 (6.1%)	3455 (3.1%)	2448 (2.6%)	1007 (5.4%)

Abuse	44 (0.2%)	25 (0.1%)	19 (0.5%)	248 (0.2%)	170 (0.2%)	78 (0.4%)

Domestic violence	31 (0.1%)	17 (<0.1%)	14 (0.3%)	274 (0.2%)	185 (0.2%)	89 (0.5%)

Parent died	736 (2.9%)	556 (2.7%)	180 (4.3%)	3145 (2.8%)	2367 (2.5%)	778 (4.2%)

Parent imprisoned	1252 (5.0%)	899 (4.3%)	353 (8.4%)	5352 (4.8%)	3917 (4.2%)	1435 (7.7%)

Parental substance abuse	2148 (8.6%)	1581 (7.6%)	567 (14%)	9559 (8.5%)	7011 (7.5%)	2548 (14%)

Parents separated	9603 (43%)	7613 (40%)	1990 (53%)	44 631 (46%)	35 422 (44%)	9209 (56%)

*Unknown*	2519	2096	423	14 749	12 488	2261

**Any SUD**	1166 (4.6%)	448 (2.1%)	718 (17%)	4867 (4.3%)	2368 (2.5%)	2499 (13%)

Cannabis	209 (0.8%)	45 (0.2%)	164 (3.9%)	446 (0.4%)	174 (0.2%)	272 (1.5%)

Opioids	92 (0.4%)	32 (0.2%)	60 (1.4%)	321 (0.3%)	122 (0.1%)	199 (1.1%)

Sedatives	165 (0.7%)	47 (0.2%)	118 (2.8%)	689 (0.6%)	203 (0.2%)	486 (2.6%)

Stimulants	178 (0.7%)	29 (0.1%)	149 (3.6%)	475 (0.4%)	185 (0.2%)	290 (1.6%)

Alcohol	670 (2.7%)	320 (1.5%)	350 (8.4%)	3405 (3.0%)	1808 (1.9%)	1597 (8.5%)

Other drugs	428 (1.7%)	85 (0.4%)	343 (8.2%)	1383 (1.2%)	499 (0.5%)	884 (4.7%)

**Birth to longest residence**						

*Always rural*	16 113 (70%)	13 508 (71%)	2605 (68%)	78 339 (73%)	65 330 (73%)	13 009 (73%)

*Rural->Urban*	590 (2.6%)	485 (2.5%)	105 (2.7%)	2278 (2.1%)	1913 (2.2%)	365 (2.1%)

*Urban->Rural*	1253 (5.5%)	1022 (5.3%)	231 (6.0%)	5292 (5.0%)	4190 (4.7%)	1102 (6.2%)

*Always urban*	5010 (22%)	4124 (22%)	886 (23%)	20 782 (19%)	17 492 (20%)	3290 (19%)

*Unknown*	2138	1781	357	5395	4480	915

N (%).

**Table 3.** Associations between the main exposures and risk of SCZ and BD (unadjusted and mutually adjusted models)

	SCZ				BD			
	Unadjusted		Adjusted		Unadjusted		Adjusted	

Exposure	IRR (95%CI)	*p*	IRR (95%CI)	*p*	IRR (95%CI)	*p*	IRR (95%CI)	*p*

Any infection	**1.10** (1.01–1.19)	0.020	1.05 (0.95–1.15)	0.34	**1.27** (1.23–1.32)	<0.001	**1.21** (1.17–1.26)	<0.001

Any ACE	**1.74** (1.62–1.86)	<0.001	**1.30** (1.19–1.42)	<0.001	**1.75** (1.69–1.80)	<0.001	**1.49** (1.44–1.55)	<0.001

Any substance	**9.94** (8.72–11.33)	<0.001	**9.43** (8.15–10.92)	<0.001	**6.11** (5.75–6.49)	<0.001	**5.50** (5.15–5.88)	<0.001

Urbanicity		0.70					0.094	

Rural → Urban	1.21 (0.96–1.51)	0.11	1.05 (0.82–1.35)	0.047	0.97 (0.86–1.09)	0.58	**0.90** (0.79–1.02)	<0.001

Urban → Rural	**1.39** (1.17–1.67)	<0.001	**1.22** (1.00–1.49)	0.020	**1.35** (1.24–1.47)	<0.001	**1.24** (1.13–1.35)	0.007

Urban → Urban	**1.34** (1.17–1.52)	<0.001	**1.19** (1.03–1.37)	0.34	0.96 (0.91–1.03)	0.24	**0.91** (0.85–0.98)	<0.001

IRR, incidence rate ratio, 95%CI, 95% confidence interval; bold indicates *p* <0.05. Reference category for binary variables is no exposure, for urbanicity is ‘rural birth and rural longest residence’. Fully adjusted model also adjusted for parental income and highest parental education.
