# Supplementary material for: Environmental risk factors for schizophrenia and bipolar disorder from childhood to diagnosis: a Swedish nested case–control study
Source: Psychol Med. 2024 Mar 1;54(9):2162–71. doi: 10.1017/S0033291724000266 (PMC11366041; doi:10.1017/S0033291724000266)
Supplement: Robinson et al. supplementary material [file S0033291724000266sup001.docx]

# Supplementary Methods

## Coding Prenatal/Maternal infections using Swedish registry data

Table S1. Infections ICD codes

| Group | Type of infection | ICD Version | Diagnostic code |
| --- | --- | --- | --- |
| Any infection | Gastrointestinal | ICD-8 | 000-009, 014, 039.92, 127.99, 522.50, 527.30, 528.00, 528.30, 540.00-540.99, 562.00, 562.19, 566.00-566.01, 567.00-567.02, 569.00, 572.99, 577.01 |
|  |  | ICD-9 | 001-009, 014, 123, 127, 129, 136E-F, 522E, 522H, 526E, 527D, 528A, 528D, 540A-X, 562-B, 566, 567-C, 569F, 575A |
|  |  | ICD-10 | A00-A09, B68, B69, B70, B71, B77-B82, B83.8, K04.4-K04.7, K10.2, K11.3, K12.1, K12.2, K35, K57, K61, K63.0, K65, K81, M02.1, T62.9 |
|  | Septicaemia | ICD-8 | 038.00-038.99, 782.9 |
|  |  | ICD-9 | 038A-X, 785F |
|  |  | ICD-10 | A40-A41 |
|  | Genitourinary | ICD-8 | 016, 054.02, 090-099, 590.00-590.99, 595.00-595.02, 597.00, 599.02, 601.00, 604.00, 604.01, 607.30, 611.00, 611.01, 612.01-614.99, 616.00-616.03, 620.00-620.99, 622.00-622.19, 629.40 |
|  |  | ICD-9 | 016, 054B, 090-099, 112B-C, 131A, 590-X, 597A, 595-X, 597W, 599A, 601-D, 603B, 604A, 604X, 607B-C, 608A-E, 611A, 614-X, 615A-X, 616-X |
|  |  | ICD-10 | A18.0-18.1, A50-64, A70-74, B37.3-37.4, N10-12, N13.6, N15.1, N15.9, N30-30.3, N30.8-30.9, N34-34.1, N39.0, N41-41.3, N43.1, N45.0-45.9, N48.1-48.2, N49-49.9, N61, N70-76.8, N98.0 |
|  | Skin infections | ICD-8 | 017.01-017.09, 050-057, 110-111, 680.00-680.90, 681.00-682.99, 684.00-684.09, 686.00-686.98 |
|  |  | ICD-9 | 017A, 031B, 050-057, 074D, 091D, 110-111, 112D, 681-682X, 683, 684, 685-686X, 680A |
|  |  | ICD-10 | A18.4, A20.0, A22.0, A26.0, A31.1, A32, A36.3, B00-B09, B35-36, B37.2, B43.0, B43.2, B45.2, B46.3, B55.1, L00-L08, L30.3, L70.0 |
|  | Viral hepatitis | ICD-8 | 070 |
|  |  | ICD-9 | 070A-X |
|  |  | ICD-10 | B15-B19 |
|  | Nervous system | ICD-8 | 013.00-013.99, 027.01, 036.00, 040.00-043.99, 045.00-046.99, 052.00, 054.04, 062.00-065.99, 071.99, 072.01, 075.02, 079.20, 084.00, 094.00-094.98, 320.00-320.80, 320.88-320.99, 322.00-322.03, 392.99, 474.99 |
|  |  | ICD-9 | 006F, 013-X, 036A-B, 045-049X, 052B, 053A, 054D, 055A, 056A, 071, 072B-C, 090E, 094-X, 320-X, 321A, 321B-H, 321W, 323A, 323C-D, 324-X, 392-X |
|  |  | ICD-10 | A06.6, A17-17.9, A20.3, A22.8, A32.1, A39.0, A80-89, B00.3-00.4, B01.0-01.1, B02.0-02.1, B05.0-05.1, B06.0, B26.1-26.2, B37.5, B38.4, B43.1, B45.1, B46.1, B50.0, B57.4, B58.2, B60.2, B69.0, B83.2, G00-00.9, G01, G02.0-02.8, G04, G04.2, G04.9, G05.0, G05.1, G05.2, G06.0-6.2, G07, I02-02.9 |
|  | Respiratory | ICD-8 | 010-012, 020.10, 460.99, 461.00-461.09, 462.01, 462.02, 462.09, 463.01, 463.09, 464.01-464.09, 465.99, 466.99, 470.99-473.99, 480.99, 481.99-482.98, 483.99-486.09, 490.99-491.09, 501.99, 502.00-503.09, 508.00-508.03, 510.01-510.09, 511.10, 513.99, 519.92 |
|  |  | ICD-9 | 010-012W, 031A, 033-034B, 052A, 055B, 112E, 122B, 460-466, 466-B, 473-X, 475, 480-X, 481-482X, 483, 485, 486, 487-W, 490, 491B, 510-X, 511B, 513-B |
|  |  | ICD-10 | A15-16, A20.2, A21.2, A22.1, A31.0, A37, A38, A48.1, B00.2, B01.2, B05.2, B27, B37.1, B39-42, B44, B45.0, B46.0, B58.3, B59, J00-J22, J32, J34.0, J35.0, J36, J37, J39.0-39.1, J40-42 |
|  | Infections associated with SCZ/BD (includes *T. gondii* (toxoplasmosis), CMV, HSV, Mumps, Influenza) | ICD-8 | 054, 072, 079.5, 130, 470-74, 761.4, 761.2 |
|  |  | ICD-9 | 054, 072, 078F, 130, 487, 760C, 771B*, 771C |
|  |  | ICD-10 | A60, B00, B25, B26, B58, J09-19, P35.1, P37.1, P352, P00.2 |
| Central nervous system infections | Includes CMV, mumps, HSV, *T. gondii* | ICD-8 | 013.00-013.99, 027.01, 036.00, 040.00-043.99, 045.00-046.99, 052.00, 054, 062.00-065.99, 071.99, 072, 075.02, 079.20, 079.5, 084.00, 094.00-094.98, 320.00-320.80, 320.88-320.99, 322.00-322.03, 392.99, 474.99, 761.4 |
|  |  | ICD-9 | 006F, 013-X, 036A-B, 045-049X, 052B, 053A, 054, 055A, 056A, 071, 072, 078F, 090E, 094-X, 320-X, 321A, 321B-H, 321W, 323A, 323C-D, 324-X, 392-X, 771B, 771C |
|  |  | ICD-10 | A06.6, A17-17.9, A20.3, A22.8, A32.1, A39.0, A60, A80-89, B00, B01.0-01.1, B02.0-02.1, B05.0-05.1, B06.0, B25, B26, B37.5, B38.4, B43.1, B45.1, B46.1, B50.0, B57.4, B58, B58.2, B60.2, B69.0, B83.2, G00-00.9, G01, G02.0-02.8, G04, G04.2, G04.9, G05.0, G05.1, G05.2, G06.0-6.2, G07, I02-02.9, P315, P352 |
| Influenza |  | ICD-8 | 470-474 |
|  |  | ICD-9 | 487 |
|  |  | ICD-10 | J09-19 |

## Coding Adverse childhood events using Swedish registry data

Table S2. Coding Individual ACEs variables

| **Register coded variables** | | | |
| --- | --- | --- | --- |
| **ACE** | Register(s) | Register years | **Variable coding** |
| Parental separation | Census | 1975, 1980, 1985, 1990 | Separated: If the parents divorced/separated between ages 0-15.  Not separated: the parents are married or cohabitating ages 0-15.  Coded as missing if the mother was single (at birth) or if a parent died when the individual was aged 0-15 |
|  | Longitudinal integrated database for health insurance and labour market studies (LISA) | 1990+ |  |
|  | Medical birth register | 1973+ |  |
| Child abuse (physical, sexual, neglect) | Crime register | 1965+ | Coded if either parent was convicted of abusing their offspring under the law 'Sexual intercourse with offspring' (1965+). |
| Parental substance abuse (drug abuse, alcohol abuse, drunk/drug driving, drug-related crime) | Crime register | 1965+ | Driving under the influence of drugs or alcohol (1951+).Drug possession (1968+). |
|  |  |  |  |
|  | Suspect register | 1975+ | Drug penalty law, holding, use, holding and use (1998+).  Drunk driving, aggravated drunk driving under the influence of alcohol alone or under the influence of both alcohol and drugs (1975+).  Driving under the influence of drugs only (2000+) |
|  |  |  |  |
|  |  |  |  |
|  |  |  |  |
|  |  |  |  |
| Parental incarceration | Sanction register | 1973+ | If either parent spent time in prison when the individual was aged 0-15 |
| Parental death | Death register | 1961+ | If either parent died when the individual was aged 0-15 |
| Violence in the home (domestic violence i.e. father/mother was convicted of abusing a partner) | National Crime Register | 1973+ | Gross violation of integrity and gross violation of a women’s integrity (1998+). |
|  | Suspect register | 1975+ | Serious violation of women's rights (1998+). Abuse, not rough or rough, indoor against woman/man 18 years of age or older in close relationship (i.e. been married, cohabits, or cohabited, or has common children) (2008+). |

| **ICD coded variables** |  |  |  |  |
| --- | --- | --- | --- | --- |
| ACE | Register | ICD8 | ICD9 | ICD10 |
| Child abuse (violence, abuse, or neglect) | National patient register | 994.4, 994.98, 996.88, 996.99, 994.2, 994.3, 994.4, (E960 - E969) | 995F, 994X, 994X, 994C, 994D, 994E, 994F, E960-E969 | T73, T74, X85-Y09 |
| Parental substance abuse (drug abuse, alcohol abuse) | National patient register | 303, 304 | 303, 304, 305 | F10, F11, F12, F13, F14, F15, F16, F16, F18, F19 |

To determine the age of the child when the crime-related ACE happened, the crime start date was used. If the start date of the crime date was missing, then the decision date was used. If there is no report of a specific ACE occurring then these are set to ‘No' i.e. if there is no reported abuse, assume that no abuse happened.

## Coding substance use disorders

Table S3. Substance abuse ICD codes

| Substance | ICD8 | ICD9 | ICD10 |
| --- | --- | --- | --- |
| Any form of substance use disorder | 303, 304 | 303, 304, 305 | F10-F16, F18, F19 |
| Cannabis | 304.5 Cannabis dependence | 304D Cannabis dependence | F12 Cannabis related disorders (excluding F12.5, psychotic Disorder Caused By Cannabis; F12.7 Psychotic disorder as residual condition or with late onset caused by cannabis) |
| Alcohol | 303 Alcoholism | 303 Alcohol dependence | F10 Alcohol related disorders |
|  |  | 305A Alcohol Abuse |  |
| Morphine/opioids | 304.0 Opioid dependence | 304A morphine and other opiates | F11 Opioid related disorders |
|  | 304.1 Morphine dependence |  |  |
| Sedatives | 304.2 Barbiturate dependence |  | F13 Sedative, hypnotic, or anxiolytic related disorders |
|  | 304.3 Tranquillisers dependence | 304B Barbiturate |  |
| Stimulants (including cocaine) | 304.6 Psychostimulant dependence | 304E Amphetamine and other psychostimulant dependence | F15 Other stimulant related disorders |
|  |  |  |  |
|  | 304.4 Cocaine dependence | 304C Cocaine dependence | F14 Cocaine related disorders |
|  |  |  |  |
| Hallucinogens | 304.7 Hallucinogen dependence | 304F Hallucinogen dependence | F16 Hallucinogen related disorders |
|  |  |  |  |
| Other drugs | 304.8 Other drug dependence | 304G Sniffing of thinner / gasoline | F18 Inhalant related disorders |
|  | 304.9 Unspecified | 304W Other drugs | F19 Other psychoactive substance related disorders |
|  |  | 304H Combinations of drugs |  |
|  |  | 304X Drug not specified |  |

*Hallucinogens were too few to study as an individual category and were excluded from analyses. In Swedish ICD9 codes there are no subcategories for non-dependent drug abuse.

## Coding urbanicity

Urbanicity was determined from population size per municipality (kommun), using data from Statistics Sweden (1968-2013)^1^. Sweden is currently divided into 290 administrative municipalities. For each year, we determined the population density as the number of inhabitants divided by the area of municipality in km^2^. The urbanicity category for each municipality was assigned according to the Eurostat definitions:

- High-density: a density of at least 1500 inhabitants per km^2^ and a minimum population of 50,000
- Urban: a density of at least 300 inhabitants per km^2^ and a minimum population of 5,000
- Rural: a density less than 300 inhabitants per km^2^

For simplicity, high density and urban were combined. The longest residence for each individual was the most frequent urbanicity category. If someone spent equivalent time in two categories, they were assigned the higher risk category (i.e. urban/high-density>rural). Each participant was classified based on population density of their municipality (i) at birth, and (ii) of longest residence prior to SCZ/BD diagnosis or sampling date, capturing complementary aspects of urban exposure (early life vs later/cumulative).

The categories were as follows:

- Always rural
- Always Urban
- Rural-->Urban
- Urban-->Rural

## Assessment of covariates

Socioeconomic status was determined from the highest achieved parental education, and highest parental disposable income when the child was age 16.

SES variables were taken from either the Labour statistics based on administrative sources 1985-1989, or from the longitudinal integrated database for health insurance and labour market studies (LISA,1990+)^2^.

Highest parental education, which utilizes the SUN2000 nomenclature (Swedish education nomenclature), was extracted when the child was aged 16. Education for both parents was categorized by compulsory, secondary, higher and postgraduate, and the highest achieved parental education was the highest level from either the biological mother or father (supplementary Table 4).

Table S4. Categorising education level in LISA

| Code start  SUN2000NIVA (OLD) | Code start  SUN2000NIVA (new) | Category | Years schooling |
| --- | --- | --- | --- |
| 1  2 | 1  2 | Compulsory (i.e. Primary and lower secondary education) | 0-9 years (compulsory schooling) |
| 3  4 | 3 | Upper secondary | 0-3 years upper secondary education |
| 5 | 4-5.3 | Higher education  (i.e. higher education/university) | 0-3 years post-secondary education |
| 6  7 | 5.4-6 | Postgraduate | 4 years post-secondary education |

Household disposable income from LISA 1990-2013 (DISPINKFAM) was determined for both parents for the years when the individual was aged 14-16. Household income was averaged (mean) from data from the three years, and the highest income (from mother/father) was stratified into quartiles based on income by year data from the controls. Due to data only being available from 1990 onwards, those born in 1973 will be missing parental income data, those born in 1974 only have data from the year they are age 16, and those born in 1975 only have data at ages 15-16.

# Supplementary RESULTS

Figure S1 Correlations between main exposure variables for the SCZ and BD matched datasets

All correlations p<0.001.

Table S5 Descriptive characteristics of the environmental risk factors in the SCZ and BD samples stratified by sex

|  | | **SCZ** | | | |  | **BD** | | | |
| --- | --- | --- | --- | --- | --- | --- | --- | --- | --- | --- |
|  | | Male | | Female | |  | Male | | Female | |
| Characteristic | | case, N = 2,780 | ctrl, N = 13,900 | case, N = 1,404 | ctrl, N = 7,020 |  | case, N = 6,306 | ctrl, N = 31,530 | case, N = 12,375 | ctrl, N = 61,875 |
| **Any infection** | | 671 (24%) | 3,111 (22%) | 286 (20%) | 1,339 (19%) |  | 1,706 (27%) | 7,455 (24%) | 3,021 (24%) | 12,269 (20%) |
| CNS infection | | 31 (1.1%) | 169 (1.2%) | 12 (0.9%) | 57 (0.8%) |  | 84 (1.3%) | 322 (1.0%) | 115 (0.9%) | 499 (0.8%) |
| Influenza | | 5 (0.2%) | 23 (0.2%) | 4 (0.3%) | 17 (0.2%) |  | 26 (0.4%) | 131 (0.4%) | 66 (0.5%) | 245 (0.4%) |
| **Any ACE** | | 1,537 (55%) | 5,748 (41%) | 729 (52%) | 2,808 (40%) |  | 3,422 (54%) | 13,311 (42%) | 6,999 (57%) | 26,232 (42%) |
| Number  of ACE*s* | *0* | 1,243 (45%) | 8,152 (59%) | 675 (48%) | 4,212 (60%) |  | 2,884 (46%) | 18,219 (58%) | 5,376 (43%) | 35,643 (58%) |
|  | *1* | 1,122 (40%) | 4,736 (34%) | 557 (40%) | 2,268 (32%) |  | 2,578 (41%) | 10,956 (35%) | 5,176 (42%) | 21,578 (35%) |
|  | *2* | 231 (8.3%) | 628 (4.5%) | 100 (7.1%) | 349 (5.0%) |  | 529 (8.4%) | 1,572 (5.0%) | 1,131 (9.1%) | 2,989 (4.8%) |
|  | *3+* | 184 (6.6%) | 384 (2.8%) | 72 (5.1%) | 191 (2.7%) |  | 315 (5.0%) | 783 (2.5%) | 692 (5.6%) | 1,665 (2.7%) |
| Abuse | | 13 (0.5%) | 14 (0.1%) | 6 (0.4%) | 11 (0.2%) |  | 19 (0.3%) | 57 (0.2%) | 59 (0.5%) | 113 (0.2%) |
| Domestic violence | | 11 (0.4%) | 13 (<0.1%) | 3 (0.2%) | 4 (<0.1%) |  | 27 (0.4%) | 55 (0.2%) | 62 (0.5%) | 130 (0.2%) |
| Parent died | | 121 (4.4%) | 367 (2.6%) | 59 (4.2%) | 189 (2.7%) |  | 292 (4.6%) | 820 (2.6%) | 486 (3.9%) | 1,547 (2.5%) |
| Parent imprisoned | | 247 (8.9%) | 590 (4.2%) | 106 (7.5%) | 309 (4.4%) |  | 454 (7.2%) | 1,268 (4.0%) | 981 (7.9%) | 2,649 (4.3%) |
| Parental substance abuse | | 395 (14%) | 1,033 (7.4%) | 172 (12%) | 548 (7.8%) |  | 812 (13%) | 2,362 (7.5%) | 1,736 (14%) | 4,649 (7.5%) |
| Parents separated | | 1,359 (54%) | 5,131 (41%) | 631 (50%) | 2,482 (40%) |  | 2,988 (54%) | 11,909 (43%) | 6,221 (57%) | 23,513 (44%) |
| **Any SUD** | | 524 (19%) | 289 (2.1%) | 194 (14%) | 159 (2.3%) |  | 973 (15%) | 889 (2.8%) | 1,526 (12%) | 1,479 (2.4%) |
| Cannabis | | 143 (5.1%) | 37 (0.3%) | 21 (1.5%) | 8 (0.1%) |  | 150 (2.4%) | 101 (0.3%) | 122 (1.0%) | 73 (0.1%) |
| Opioids | | 45 (1.6%) | 24 (0.2%) | 15 (1.1%) | 8 (0.1%) |  | 90 (1.4%) | 61 (0.2%) | 109 (0.9%) | 61 (<0.1%) |
| Sedatives | | 76 (2.7%) | 26 (0.2%) | 42 (3.0%) | 21 (0.3%) |  | 153 (2.4%) | 78 (0.2%) | 333 (2.7%) | 125 (0.2%) |
| Stimulants | | 106 (3.8%) | 15 (0.1%) | 43 (3.1%) | 14 (0.2%) |  | 121 (1.9%) | 85 (0.3%) | 169 (1.4%) | 100 (0.2%) |
| Alcohol | | 250 (9.0%) | 210 (1.5%) | 100 (7.1%) | 110 (1.6%) |  | 595 (9.4%) | 635 (2.0%) | 1,002 (8.1%) | 1,173 (1.9%) |
| Other drugs | | 252 (9.1%) | 57 (0.4%) | 91 (6.5%) | 28 (0.4%) |  | 387 (6.1%) | 229 (0.7%) | 497 (4.0%) | 270 (0.4%) |
| **Birth to longest residence** | |  |  |  |  |  |  |  |  |  |
| *Always rural* | | 1,741 (68%) | 9,055 (71%) | 864 (67%) | 4,453 (69%) |  | 4,310 (72%) | 21,838 (73%) | 8,699 (74%) | 43,492 (74%) |
| *Rural->Urban* | | 68 (2.7%) | 322 (2.5%) | 37 (2.9%) | 163 (2.5%) |  | 146 (2.4%) | 664 (2.2%) | 219 (1.9%) | 1,249 (2.1%) |
| *Urban->Rural* | | 152 (6.0%) | 645 (5.1%) | 79 (6.2%) | 377 (5.9%) |  | 404 (6.8%) | 1,450 (4.9%) | 698 (5.9%) | 2,740 (4.6%) |
| *Always urban* | | 585 (23%) | 2,704 (21%) | 301 (23%) | 1,420 (22%) |  | 1,114 (19%) | 5,930 (20%) | 2,176 (18%) | 11,562 (20%) |

N (%) exposed.

Table S6. Unadjusted conditional logistic regression results

|  |  | **SCZ** | | | | **BD** | | | | **Wald test** | | |
| --- | --- | --- | --- | --- | --- | --- | --- | --- | --- | --- | --- | --- |
| Group | Exposure | IRR | 95%CI | p | p_FDR_ | IRR | 95%CI | p | p_FDR_ | Diff | 95% CI | p |
| Infections  0-15yrs | Any infection | **1.10** | (1.01-1.19) | 0.020 | 0.023 | **1.27** | (1.23-1.32) | <0.001 | <0.001 | **0.86** | (0.79-0.94) | 0.001 |
|  | CNS infection | 0.95 | (0.69-1.32) | 0.76 | 0.763 | **1.22** | (1.04-1.42) | 0.014 | 0.018 | 0.78 | (0.54-1.12) | 0.19 |
|  | Influenza | 1.13 | (0.55-2.32) | 0.75 | 0.763 | 1.23 | (0.98-1.54) | 0.081 | 0.090 | 0.92 | (0.43-1.96) | 0.82 |
| ACEs  0-15yrs | Any ACE | **1.74** | (1.62-1.86) | <0.001 | <0.001 | **1.75** | (1.69-1.80) | <0.001 | <0.001 | 0.99 | (0.92-1.07) | 0.89 |
|  | 1 ACE | **1.57** | (1.46-1.69) | <0.001 | <0.001 | **1.58** | (1.52-1.63) | <0.001 | <0.001 | 0.99 | (0.92-1.08) | 0.88 |
|  | 2 ACES | **2.21** | (1.93-2.53) | <0.001 | <0.001 | **2.40** | (2.26-2.55) | <0.001 | <0.001 | 0.92 | (0.79-1.07) | 0.27 |
|  | 3+ ACEs | **2.86** | (2.45-3.34) | <0.001 | <0.001 | **2.71** | (2.51-2.93) | <0.001 | <0.001 | 1.06 | (0.89-1.26) | 0.53 |
|  | Abuse | **3.80** | (2.09-6.90) | <0.001 | <0.001 | **2.31** | (1.77-3.03) | <0.001 | <0.001 | 1.64 | (0.85-3.16) | 0.14 |
|  | Domestic violence | **4.25** | (2.07-8.72) | <0.001 | <0.001 | **2.43** | (1.88-3.13) | <0.001 | <0.001 | 1.75 | (0.82-3.76) | 0.15 |
|  | Parental death | **1.65** | (1.39-1.96) | <0.001 | <0.001 | **1.67** | (1.54-1.82) | <0.001 | <0.001 | 0.99 | (0.82-1.20) | 0.90 |
|  | Parental prison | **2.06** | (1.81-2.34) | <0.001 | <0.001 | **1.90** | (1.79-2.03) | <0.001 | <0.001 | 1.08 | (0.94-1.25) | 0.29 |
|  | Parental substance abuse | **1.92** | (1.73-2.12) | <0.001 | <0.001 | **1.95** | (1.86-2.05) | <0.001 | <0.001 | 0.98 | (0.88-1.10) | 0.76 |
|  | Parental separation | **1.69** | (1.57-1.82) | <0.001 | <0.001 | **1.65** | (1.59-1.71) | <0.001 | <0.001 | 1.02 | (0.95-1.11) | 0.55 |
| Substance use disorder | Any substance | **9.94** | (8.72-11.33) | <0.001 | <0.001 | **6.11** | (5.75-6.49) | <0.001 | <0.001 | **1.63** | (1.41-1.88) | <0.001 |
|  | Cannabis | **19.33** | (13.77-27.13) | <0.001 | <0.001 | **8.08** | (6.66-9.80) | <0.001 | <0.001 | **2.39** | (1.62-3.54) | <0.001 |
|  | Opioids | **10.09** | (6.47-15.73) | <0.001 | <0.001 | **8.20** | (6.54-10.28) | <0.001 | <0.001 | 1.23 | (0.75-2.02) | 0.42 |
|  | Sedatives | **13.23** | (9.36-18.71) | <0.001 | <0.001 | **12.31** | (10.42-14.53) | <0.001 | <0.001 | 1.08 | (0.73-1.58) | 0.71 |
|  | Stimulants | **26.51** | (17.70-39.69) | <0.001 | <0.001 | **8.08** | (6.70-9.75) | <0.001 | <0.001 | **3.28** | (2.10-5.12) | <0.001 |
|  | Alcohol | **6.05** | (5.16-7.09) | <0.001 | <0.001 | **4.86** | (4.53-5.21) | <0.001 | <0.001 | **1.25** | (1.05-1.48) | 0.014 |
|  | Other drugs | **24.21** | (18.69-31.37) | <0.001 | <0.001 | **9.42** | (8.41-10.54) | <0.001 | <0.001 | **2.57** | (1.94-3.41) | <0.001 |
| Birth to longest residence | Rural🡪urban | 1.21 | (0.96-1.51) | 0.10 | 0.11 | 0.97 | (0.86-1.09) | 0.58 | 0.601 | 1.25 | (0.97-1.60) | 0.086 |
|  | Urban🡪rural | **1.39** | (1.17-1.67) | <0.001 | <0.001 | **1.35** | (1.24-1.47) | <0.001 | <0.001 | 1.03 | (0.85-1.25) | 0.75 |
|  | Urban🡪urban | **1.34** | (1.17-1.52) | <0.001 | <0.001 | 0.96 | (0.91-1.03) | 0.24 | 0.258 | **1.39** | (1.20-1.60) | <0.001 |

IRR, Incidence rate ratio; CI ,Confidence Interval; p, p-value; pFDR, False discovery rate p value. Differences in IRR between SCZ and BD evaluated using Wald-tests. Regression model estimates are relative to 'no exposure', or ‘rural birth & rural longest residence’ for urbanicity.

Table S7 sex stratified models for the Associations between the main exposures and risk of SCZ and BD (unadjusted and mutually adjusted models)

| Adjusted | **SCZ** | | | | | | |  | | **BD** | | | | | | | |
| --- | --- | --- | --- | --- | --- | --- | --- | --- | --- | --- | --- | --- | --- | --- | --- | --- | --- |
|  | Female | | |  | Male | | |  |  | Female | | |  | Male | | |  |
| Exposure | aIRR | 95%CI | pvalue |  | aIRR | 95%CI | pvalue |  |  | aIRR | 95%CI | pvalue |  | aIRR | 95%CI | pvalue |  |
| Any infection | 1.00 | (0.84, 1.17) | 0.961 |  | 1.07 | (0.96, 1.20) | 0.235 |  |  | 1.25 | (1.19, 1.32) | **<0.001** |  | 1.14 | (1.06, 1.22) | **<0.001** |  |
| Any ACE | 1.25 | (1.08, 1.44) | **0.003** |  | 1.33 | (1.20, 1.48) | **<0.001** |  |  | 1.53 | (1.46, 1.60) | **<0.001** |  | 1.41 | (1.32, 1.51) | **<0.001** |  |
| Any substance | 7.25 | (5.63, 9.35) | **<0.001** |  | 10.78 | (9.00, 12.93) | **<0.001** |  |  | 5.24 | (4.82, 5.69) | **<0.001** |  | 6.05 | (5.43, 6.75) | **<0.001** |  |
| Rural->urban | 1.12 | (0.74, 1.68) | 0.602 |  | 1.01 | (0.74, 1.39) | 0.926 |  |  | 0.82 | (0.70, 0.96) | **0.015** |  | 1.05 | (0.86, 1.28) | 0.639 |  |
| Urban->rural | 1.03 | (0.74, 1.43) | 0.871 |  | 1.35 | (1.06, 1.73) | **0.017** |  |  | 1.17 | (1.05, 1.31) | **0.005** |  | 1.38 | (1.19, 1.61) | **<0.001** |  |
| Urban->urban | 1.15 | (0.90, 1.46) | 0.256 |  | 1.20 | (1.01, 1.44) | **0.043** |  |  | 0.90 | (0.83, 0.98) | **0.011** |  | 0.94 | (0.84, 1.06) | 0.297 |  |

aIRR, adjusted IRR. 95%CI, 95% Confidence Interval; Bold indicates p<0.05. Reference category for binary variables is no exposure, for urbanicity is ‘rural birth & rural longest residence’.
Fully adjusted model also adjusted for parental income and highest parental education (age 14-16).

Table S8 IRR FOR THE INDIVIDUAL ENVIRONMENTAL EXPOSURES AND RISK OF SCZ AND BD, stratified by sex.

| **SCZ** | | | | | | | | | | | |
| --- | --- | --- | --- | --- | --- | --- | --- | --- | --- | --- | --- |
|  | Female | | |  | Male | | |  |  |  | Wald test |
| Exposure | IRR | CI | pvalue |  | IRR | CI | pvalue |  | IRR-ratio | CI | pValue |
| Any infection | 1.09 | ( 0.94- 1.26) | 0.255 |  | 1.11 | ( 1.00- 1.22) | 0.042 |  | 1.02 | (0.85, 1.21) | 0.857 |
| CNS infection | 1.05 | ( 0.56- 1.96) | 0.872 |  | 0.92 | ( 0.62- 1.35) | 0.656 |  | 0.87 | (0.42, 1.81) | 0.71 |
| Influenza | 1.18 | ( 0.40- 3.50) | 0.770 |  | 1.09 | ( 0.41- 2.86) | 0.866 |  | 0.92 | (0.22, 3.96) | 0.915 |
| Any ACE | 1.64 | ( 1.46- 1.85) | **<0.001** |  | 1.79 | ( 1.64- 1.94) | <0.001 |  | 1.09 | (0.94, 1.26) | 0.254 |
| 1 ACE | 1.55 | ( 1.37- 1.76) | **<0.001** |  | 1.58 | ( 1.44- 1.72) | <0.001 |  | 1.02 | (0.87, 1.19) | 0.844 |
| 2 ACES | 1.81 | ( 1.42- 2.29) | **<0.001** |  | 2.45 | ( 2.08- 2.88) | <0.001 |  | 1.35 | (1.01, 1.81) | **0.04** |
| 3+ ACEs | 2.34 | ( 1.76- 3.10) | **<0.001** |  | 3.15 | ( 2.61- 3.79) | <0.001 |  | 1.35 | (0.96, 1.89) | 0.085 |
| Abuse | 2.73 | ( 1.01- 7.37) | **0.048** |  | 4.64 | ( 2.18- 9.88) | <0.001 |  | 1.70 | (0.49, 5.93) | 0.404 |
| Domestic violence | 3.75 | ( 0.84-16.76) | 0.084 |  | 4.41 | ( 1.94-10.03) | <0.001 |  | 1.18 | (0.21, 6.49) | 0.852 |
| Parental death | 1.59 | ( 1.18- 2.15) | **0.002** |  | 1.68 | ( 1.36- 2.08) | <0.001 |  | 1.06 | (0.73, 1.52) | 0.769 |
| Parental prison | 1.78 | ( 1.42- 2.24) | **<0.001** |  | 2.20 | ( 1.89- 2.57) | <0.001 |  | 1.24 | (0.94, 1.63) | 0.132 |
| Parental substance abuse | 1.64 | ( 1.37- 1.97) | **<0.001** |  | 2.07 | ( 1.82- 2.34) | <0.001 |  | 1.26 | (1.01, 1.56) | **0.042** |
| Parental separation | 1.53 | ( 1.35- 1.74) | **<0.001** |  | 1.77 | ( 1.62- 1.94) | <0.001 |  | 1.15 | (0.99, 1.35) | 0.068 |
| Any substance | 6.99 | ( 5.58- 8.74) | **<0.001** |  | 11.80 | (10.03-13.88) | <0.001 |  | 0.95 | (0.59, 1.51) | **<0.001** |
| Cannabis | 13.12 | ( 5.81-29.63) | **<0.001** |  | 20.79 | (14.30-30.22) | <0.001 |  | 1.17 | (0.80, 1.69) | 0.315 |
| Opioids | 9.37 | ( 3.97-22.11) | **<0.001** |  | 10.36 | ( 6.16-17.41) | <0.001 |  | 1.07 | (0.81, 1.40) | 0.846 |
| Sedatives | 10.37 | ( 6.09-17.68) | **<0.001** |  | 15.61 | ( 9.86-24.71) | <0.001 |  | 1.69 | (1.28, 2.23) | 0.255 |
| Stimulants | 15.36 | ( 8.40-28.07) | **<0.001** |  | 37.65 | (21.56-65.75) | <0.001 |  | 1.58 | (0.65, 3.88) | **0.032** |
| Alcohol | 4.91 | ( 3.70- 6.51) | **<0.001** |  | 6.67 | ( 5.49- 8.10) | <0.001 |  | 1.10 | (0.41, 3.01) | 0.079 |
| Other drugs | 17.29 | (11.18-26.75) | **<0.001** |  | 28.39 | (20.54-39.25) | <0.001 |  | 1.50 | (0.74, 3.04) | 0.074 |
| Rural🡪urban | 1.25 | ( 0.85- 1.83) | 0.250 |  | 1.18 | ( 0.90- 1.56) | 0.235 |  | 2.45 | (1.08, 5.57) | 0.817 |
| Urban🡪rural | 1.26 | ( 0.93- 1.71) | 0.133 |  | 1.47 | ( 1.18- 1.83) | <0.001 |  | 1.36 | (0.97, 1.91) | 0.418 |
| Urban🡪urban | 1.28 | ( 1.02- 1.60) | **0.030** |  | 1.37 | ( 1.16- 1.60) | <0.001 |  | 1.64 | (0.95, 2.83) | 0.64 |

| **BD** | | | | | | | | | | | |
| --- | --- | --- | --- | --- | --- | --- | --- | --- | --- | --- | --- |
|  | Female | | |  | Male | | |  |  |  | Wald test |
| Exposure | IRR | CI | pvalue |  | IRR | CI | pvalue |  | IRR-ratio | CI | pValue |
| Any infection | 1.31 | ( 1.25- 1.37) | **<0.001** |  | 1.20 | (1.13- 1.28) | **<0.001** |  | 0.92 | (0.85, 0.99) | **0.028** |
| CNS infection | 1.15 | ( 0.94- 1.42) | 0.167 |  | 1.31 | (1.03- 1.67) | **0.029** |  | 1.13 | (0.83, 1.55) | 0.439 |
| Influenza | 1.35 | ( 1.03- 1.78) | 0.030 |  | 0.99 | (0.65- 1.51) | 0.971 |  | 0.73 | (0.44, 1.21) | 0.227 |
| Any ACE | 1.80 | ( 1.73- 1.87) | **<0.001** |  | 1.65 | (1.56- 1.75) | **<0.001** |  | 0.92 | (0.86, 0.98) | **0.016** |
| 1 ACE | 1.61 | ( 1.55- 1.68) | **<0.001** |  | 1.51 | (1.42- 1.60) | **<0.001** |  | 0.94 | (0.87, 1.01) | 0.078 |
| 2 ACES | 2.53 | ( 2.35- 2.73) | **<0.001** |  | 2.16 | (1.94- 2.40) | **<0.001** |  | 0.85 | (0.75, 0.97) | **0.017** |
| 3+ ACEs | 2.78 | ( 2.53- 3.05) | **<0.001** |  | 2.58 | (2.25- 2.95) | **<0.001** |  | 0.93 | (0.78, 1.09) | 0.368 |
| Abuse | 2.63 | ( 1.91- 3.60) | **<0.001** |  | 1.68 | (1.00- 2.85) | 0.052 |  | 0.64 | (0.35, 1.18) | 0.155 |
| Domestic violence | 2.41 | ( 1.78- 3.27) | **<0.001** |  | 2.47 | (1.55- 3.92) | **<0.001** |  | 1.03 | (0.59, 1.78) | 0.929 |
| Parental death | 1.60 | ( 1.44- 1.77) | **<0.001** |  | 1.82 | (1.59- 2.09) | **<0.001** |  | 1.14 | (0.96, 1.35) | 0.136 |
| Parental prison | 1.93 | ( 1.78- 2.08) | **<0.001** |  | 1.86 | (1.66- 2.08) | **<0.001** |  | 0.96 | (0.84, 1.10) | 0.599 |
| Parental substance abuse | 2.01 | ( 1.90- 2.14) | **<0.001** |  | 1.83 | (1.68- 1.99) | **<0.001** |  | 0.91 | (0.82, 1.01) | 0.074 |
| Parental separation | 1.70 | ( 1.63- 1.78) | **<0.001** |  | 1.55 | (1.46- 1.64) | **<0.001** |  | 0.91 | (0.84, 0.98) | **0.01** |
| Any substance | 5.90 | ( 5.46- 6.37) | **<0.001** |  | 6.47 | (5.86- 7.14) | **<0.001** |  | 1.31 | (1.03, 1.66) | 0.149 |
| Cannabis | 8.44 | ( 6.31-11.29) | **<0.001** |  | 7.80 | (6.02-10.10) | **<0.001** |  | 1.17 | (0.99, 1.40) | 0.693 |
| Opioids | 8.93 | ( 6.53-12.22) | **<0.001** |  | 7.46 | (5.38-10.34) | **<0.001** |  | 1.07 | (0.94, 1.22) | 0.435 |
| Sedatives | 13.95 | (11.31-17.22) | **<0.001** |  | 9.81 | (7.47-12.88) | **<0.001** |  | 1.10 | (0.97, 1.24) | **0.045** |
| Stimulants | 8.64 | ( 6.73-11.09) | **<0.001** |  | 7.41 | (5.59- 9.84) | **<0.001** |  | 0.92 | (0.63, 1.36) | 0.429 |
| Alcohol | 4.68 | ( 4.28- 5.11) | **<0.001** |  | 5.19 | (4.61- 5.84) | **<0.001** |  | 0.84 | (0.53, 1.31) | 0.169 |
| Other drugs | 9.71 | ( 8.34-11.31) | **<0.001** |  | 9.06 | (7.65-10.73) | **<0.001** |  | 0.70 | (0.50, 0.99) | 0.55 |
| Rural🡪urban | 0.88 | ( 0.76- 1.02) | 0.083 |  | 1.15 | (0.95- 1.39) | 0.147 |  | 0.86 | (0.59, 1.25) | **0.027** |
| Urban🡪rural | 1.28 | ( 1.15- 1.42) | **<0.001** |  | 1.50 | (1.31- 1.73) | **<0.001** |  | 1.11 | (0.96, 1.29) | 0.069 |
| Urban🡪urban | 0.94 | ( 0.87- 1.02) | 0.124 |  | 1.01 | (0.90- 1.12) | 0.880 |  | 0.93 | (0.74, 1.17) | 0.312 |

_IRR, Incidence rate ratio, CI, Confidence Interval; Bold indicates p<0.05. Wald tests were used to evaluate sex differences in the estimates._


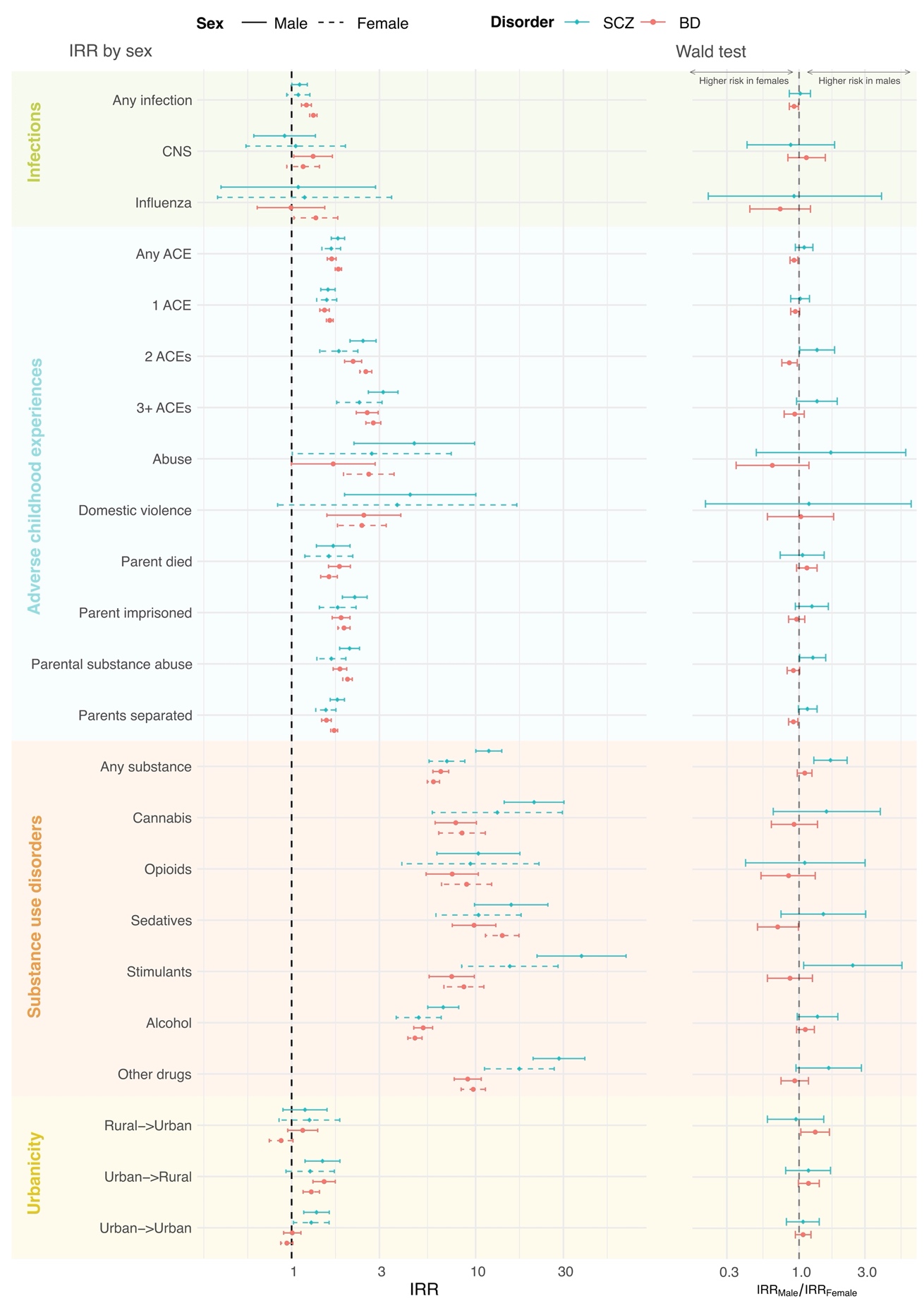


Figure s2 IRR FOR THE INDIVIDUAL ENVIRONMENTAL EXPOSURES AND RISK OF SCZ AND BD stratified by sex.
The left hand panel shows the unadjusted risk estimates and 95% confidence intervals for each exposure. The right hand panel shows the comparison of the effects for males and females; estimates greater than 1 are higher in males, and less than 1 are higher in females. axes are on the log scale.

## References

1. SCB (2022). Folkmängden i Sveriges kommuner 1950-2021. Retrieved from: <https://www.scb.se/hitta-statistik/statistik-efter-amne/befolkning/befolkningens-sammansattning/befolkningsstatistik/pong/tabell-och-diagram/helarsstatistik--kommun-lan-och-riket/folkmangden-i-sveriges-kommuner-19502021-enligt-indelning-1-januari-2022/>

2. Ludvigsson JF, Svedberg P, Olén O, Bruze G, Neovius M. The longitudinal integrated database for health insurance and labour market studies (LISA) and its use in medical research. *Eur J Epidemiol.* 2019;34(4):423-437
